# Non-typhoidal Salmonella Infections: Insights From a Case With Cardiac and Bone Involvement and Multisystem Complications

**DOI:** 10.7759/cureus.80940

**Published:** 2025-03-21

**Authors:** Ana Santos E Silva, Filipe Dias, Margarida Santos, Monica Ferro Silva, Josiana Duarte

**Affiliations:** 1 Department of Internal Medicine, Unidade Local de Saúde do Litoral Alentejano, Santiago do Cacém, PRT

**Keywords:** cervical spondylodiscitis, endocarditis, renal abscess, salmonella-induced gastroenteritis, salmonella infection

## Abstract

Non-typhoidal *Salmonella* is commonly associated with diarrhea and is generally considered a mild illness. However, in vulnerable individuals, the infection can progress to more severe conditions, including bacteremia and multisystem involvement. Invasive *Salmonella* disease presents a significant challenge for clinicians, not only because of its clinical severity but also due to its rarity and the lack of established guidelines. This case report discusses a 61-year-old woman with multiple comorbidities who developed a complex clinical condition, progressing to endocarditis with septic embolization. The diagnostic evaluation uncovered a renal abscess, spondylodiscitis, and suspected valvular vegetations linked to systemic embolization.

## Introduction

Non-typhoidal *Salmonella* (NTS) is a common cause of diarrheal illness, typically transmitted through contaminated food or water [1-4]. Infections with *Salmonella enterica* often present with fever, diarrhea, and cramping abdominal pain [2,3,5]. Although generally considered a benign condition, more vulnerable patients can develop severe complications, most frequently associated with bacteremia [1-4].

Bacteremia is an uncommon complication of *Salmonella* gastroenteritis, occurring in approximately 5% of cases, with a higher risk in immunocompromised individuals [2,3,6]. Treatment of these patients has become increasingly challenging due to the rising incidence of strains resistant to levofloxacin and cephalosporins, which are commonly used as first-line therapies [7].

While hematogenous dissemination of *S. enterica* can potentially affect any anatomical site, focal infectious metastasis to bones, meninges, or lungs remains rare. However, the cardiovascular system, despite its rarity, is the most commonly targeted site of metastatic infection [2]. Cardiovascular infections have a mortality rate of approximately 15%, with* S. enterica *serovars Typhimurium and Enteritidis responsible for over 80% of invasive cases [1].

Cardiovascular infections caused by *S. enterica *are uncommon, and their clinical characteristics, prognosis, and optimal treatment strategies remain poorly defined [2,4]. Unlike most gram-negative bacteria, *S. enterica* exhibits a unique ability to adhere to damaged endothelial cells, particularly within the heart and arterial walls, leading to conditions such as mycotic aneurysms and endocarditis [2]. *Salmonella* infective endocarditis is rare but severe, with high mortality rates due to the risk of valve perforation and leaflet rupture [7].

UTIs caused by NTS species are infrequent, with a reported prevalence of 0.015-0.9% in urinary isolates [3,8]. Such infections may result from hematogenous dissemination or direct invasion through the urethra [8]. Clinical manifestations resemble those of typical UTIs, including pyelonephritis, cystitis, and prostatitis [3].

Osteomyelitis and epidural abscesses are rare complications of *Salmonella* infection, accounting for approximately 0.8% of all cases [5,9]. Although relatively uncommon in Western populations, these conditions carry significant morbidity and mortality and should be considered in the differential diagnosis of patients presenting with prolonged fever or a history of diarrheal illness [5,9].

## Case presentation

A 61-year-old autonomous woman with a history of poorly controlled type 2 diabetes mellitus (non-insulin-treated), valvular heart failure with reduced ejection fraction, poorly managed arterial hypertension, and dyslipidemia with suboptimal metabolic control. In 2022, she underwent mechanical valve replacement for aortic and mitral insufficiency.

The patient had been previously asymptomatic but presented with acute *Salmonella* gastroenteritis requiring hospitalization. After five days of empirical antibiotic therapy with levofloxacin, she was discharged. Following discharge, her symptoms progressively worsened, with complaints of asthenia, neck pain, and epistaxis, prompting her to visit the emergency department one week after the onset of her initial condition.

An initial evaluation with spinal CT suggested osteoarticular pathology, leading to a referral for orthopedic consultation. Further investigations revealed elevated inflammatory markers and urinalysis showing leukocyturia, resulting in a presumptive diagnosis of a UTI and the initiation of empirical antibiotic therapy with amoxicillin and clavulanic acid.

Three days later, the patient returned to the emergency department due to persistent cervical pain, new-onset lumbar pain, fever, and reported dyspnea. Upon admission, a physical examination revealed a questionable bilateral renal Murphy sign (a clinical test used to detect kidney inflammation), while the neurological examination remained unremarkable.

Laboratory results indicated elevated inflammatory markers, along with evidence of hypoxemia and hypocapnia. The complete laboratory data, including trends over time and reference ranges, are provided in Table 1. Notably, acute kidney injury was present at admission, with a maximum creatinine level of 3.9 mg/dL. However, this improved throughout hospitalization, accompanied by a progressive decrease in inflammatory parameters (CRP and procalcitonin) during the entire hospital stay.

**Table 1:** Evolution of analytical markers from admission through hospitalization LDH, lactate dehydrogenase; NT-proBNP, N-terminal pro-B-type natriuretic peptide

Parameter	Reference values	D0 (initial)	D5	D7	D10 (post-transfusion)	D17	D24	D49	Discharge
Hemoglobin (g/dL)	11.7-15.5	8.2	8.2	6.6	8.4	8.6	7.8	9	10.2
Leukocytes (× 10³/uL)	4.0-11.0	10.5	15.8	13.7	18.1	6.8	5	4.4	4.7
Neutrophils (× 10³/uL)	1.6-8.3	9.7	13.6	10.8	15.6	4.3	2.6	2	2.9
Platelets (× 10³/uL)	150-400	456	554	573	644	467	309	401	306
Activated partial thromboplastin time (s)	23.8-35.8	35.6	59.6	-	32.4	34.8	36.7	45.4	38
Glucose (mg/dL)	74-106	467	220	-	140	-	89	-	-
Urea (mg/dL)	<43	74	131	106	74	43	40	66	68
Creatinine (mg/dL)	0.7-1.1	1.4	3.9	2.7	1.7	0.8	0.9	1.5	1.4
Sodium (mmol/L)	136-146	128	133	134	135	128	124	139	142
Potassium (mmol/L)	3.5-5.1	5.4	6.1	4.7	4.6	4.8	4.9	4.9	5
LDH (UI/L)	25-248	262	304	218	283	-	155	-	252
CRP (mg/dL)	<0.50	33.66	22.5	15.6	17.33	6.23	6.09	5.5	1.5
Procalcitonin (ng/mL)	<0.5	-	2.25	1.61	0.46	-	0.08	0.08	-
NT-proBNP (pg/mL)	<900	-	-	-	-	-	4411	-	-

**Figure 1:**
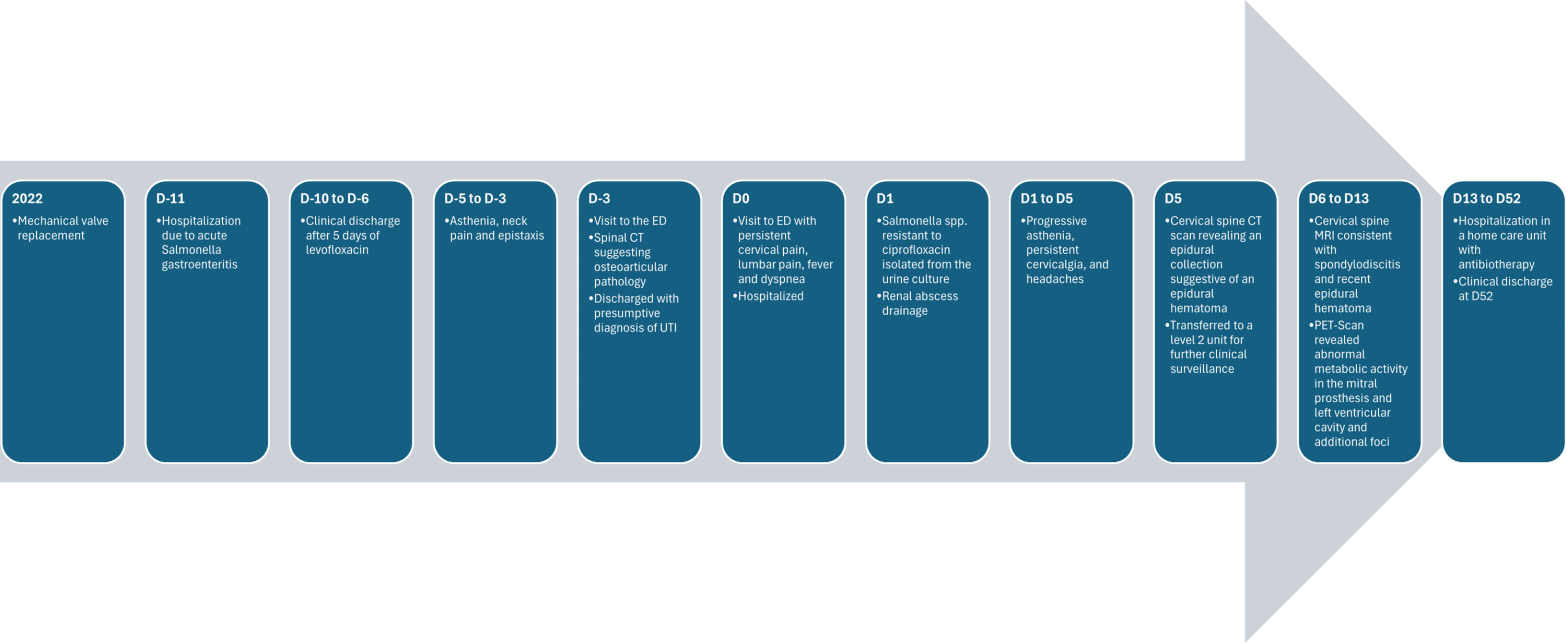
Timeline illustrating the main events in the patient’s clinical evolution

A thoracic angiographic CT scan was performed, which excluded pulmonary embolism but revealed moderate bilateral pleural effusion and a hypodense area in the right kidney suggestive of a renal abscess. Due to the suspicion of pyonephrosis, urine and blood cultures were obtained, and empiric therapy with piperacillin + tazobactam was initiated. The patient was subsequently hospitalized for further management. Figure 1 shows a timeline of the main events in the patient’s clinical course.

On the first day of hospitalization, *Salmonella* spp. resistant to ciprofloxacin was isolated from the urine culture, prompting the patient’s transfer to a reference center for renal abscess drainage. Purulent content was evacuated, and the patient returned to the original hospital for continued antibiotic therapy. During hospitalization, the patient demonstrated improvement in inflammatory markers and maintained apyrexia. *Salmonella* enteritidis was later isolated from urine, blood cultures, and renal abscess drainage fluid.

Neurologically, the patient exhibited progressive asthenia, persistent cervicalgia, and headaches. Due to neurological deterioration and ongoing neck pain, a head and cervical spine CT scan was performed, revealing an epidural collection suggestive of an epidural hematoma. Following the neurosurgery team's recommendations, a cervical spine MRI was obtained to provide further diagnostic insight and guide management.

The patient was then transferred to a level 2 unit for closer clinical monitoring. The cervical spine MRI demonstrated inflammatory infiltrate in the spinal cord, extending to the odontoid process, along with linear hypointensities consistent with spondylodiscitis and a recent epidural hematoma. Based on these findings, the case was reevaluated with the neurosurgery team, who recommended the use of a Jewett brace and continued neurological monitoring. Considering the location of the findings and the absence of neurological deficits, surgical intervention was deemed unnecessary after a careful assessment of risks and benefits.

**Figure 2:**
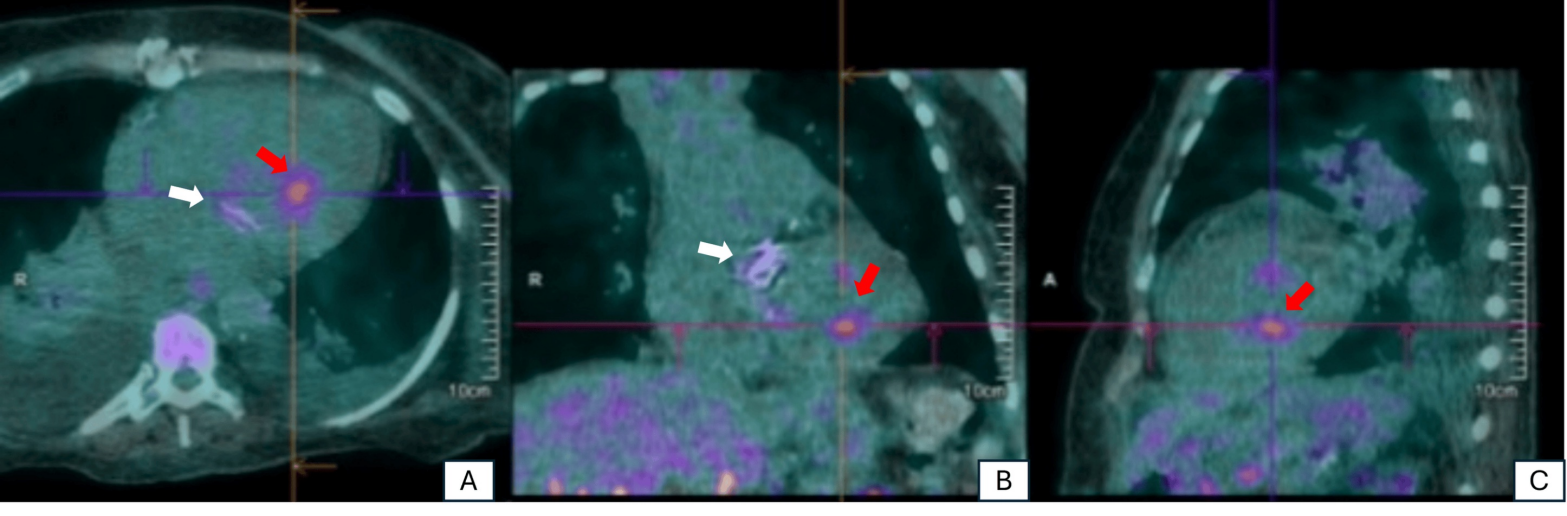
PET scan showing evidence of cardiac involvement due to Salmonella infection: (A) axial section; (B) coronal section; and (C) sagittal section The white arrow indicates prosthetic mitral valve involvement, while the red arrow highlights myocardial involvement.

**Figure 3:**
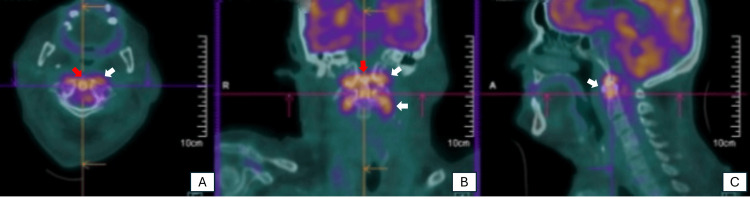
PET scan demonstrating the involvement of the C1 and C2 vertebrae consistent with Salmonella spondylodiscitis: (A) axial section; (B) coronal section; and (C) sagittal section The white arrow indicates the involvement of the C1 and C2 vertebrae, while the red arrow indicates the involvement of the odontoid process.

**Figure 4:**
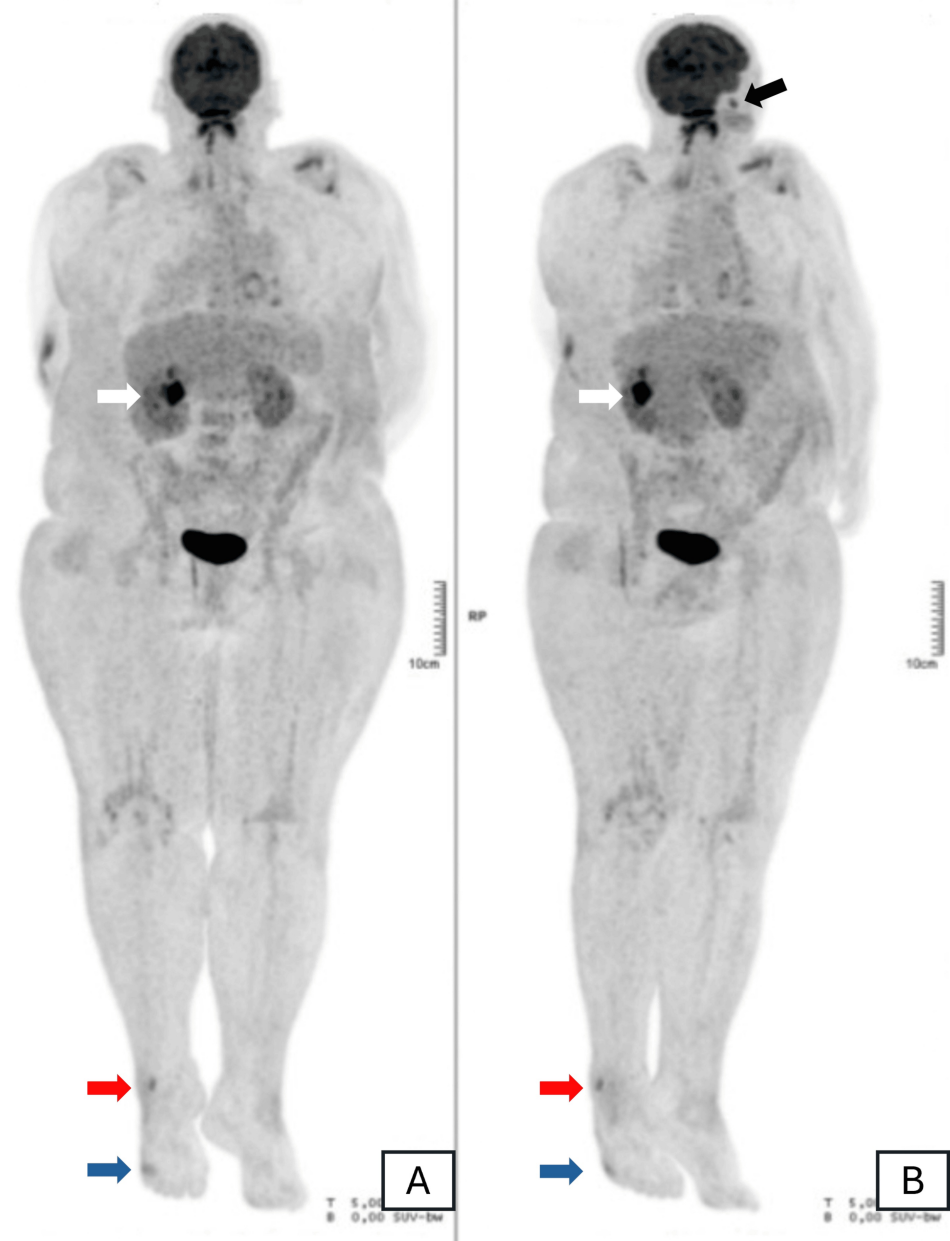
PET scan in maximum intensity projection image demonstrating multiple foci of hypermetabolism suggestive of septic embolization: (A) normal anatomical positioning and (B) normal anatomical positioning with head rotation The white arrow indicates right kidney involvement, the black arrow indicates left pterygoid muscle involvement, the red arrow indicates right fibula involvement, and the blue arrow indicates right fifth toe involvement.

Given the history of *Salmonella*-induced gastroenteritis, bacterial endocarditis was suspected, prompting further diagnostic evaluations to investigate potential septic embolization. Transthoracic echocardiography revealed no significant valvular alterations. However, a PET scan showed abnormal metabolic activity in the mitral prosthesis and left ventricular cavity, suggestive of valvular vegetations (Figure 2). As previously indicated by CT, the PET scan further confirmed vertebral involvement in the cervical spine (Figure 3). Additional areas of hypermetabolism were identified in the posterior aspect of the mid-right kidney, left pterygoid muscle, right fibula, and the base of the right fifth toe, indicating potential septic emboli (Figure 4).

In light of the evidence of non-native valve endocarditis, the patient’s management plan was discussed with the cardiology and cardiac surgery teams previously involved in her care. It was decided to continue antibiotic therapy for at least six weeks, with an imaging reevaluation planned before completing the regimen. This approach aimed to assess the need for valve replacement, ensure adequate focus control, and determine whether an extension of antibiotic therapy was necessary.

The clinical presentation was consistent with infectious endocarditis of a non-native valve, complicated by septic embolization resulting in renal abscess, spondylodiscitis, osteomyelitis, and muscular embolization.

Given the severity of the condition, the decision was made to continue antibiotic therapy under hospitalization within a home care unit to ensure the prompt detection of potential complications. Regarding spondylodiscitis, the favorable clinical progression supported the decision to avoid surgical intervention. From a cardiac standpoint, in the absence of prosthetic valve dysfunction, antibiotic therapy was maintained. The patient was scheduled for outpatient follow-up with the cardiology team while awaiting a multidisciplinary decision to determine the optimal timing for valve replacement.

## Discussion

NTS is a gram-negative bacillus and a leading cause of diarrhea worldwide [3]. Thousands of human salmonellosis cases are reported annually in Europe, with over 95% of infections resulting from foodborne transmission [2,3]. Interpersonal transmission, whether community acquired or nosocomial, is rare due to the short duration of bacterial shedding [2,3].

While the clinical presentation of NTS infection varies, most cases are mild and self-limiting without complications [2-4,6]. However, children, the elderly, and immunocompromised individuals may develop severe complications, potentially leading to a fulminant course and fatal outcomes, usually due to bacteremia [1-4]. Approximately 5% of immunocompetent patients experience bacteremia during the gastrointestinal phase [7]. Although bacteremia often follows gastrointestinal infection, some patients may present with invasive disease as the initial manifestation, characterized by a non-specific febrile illness without a history of diarrhea, complicating diagnosis [1].

The risk of bacteremia increases to 25% in individuals over 50 years of age, as *Salmonella* exhibits a preference for damaged or atherosclerotic endothelium, heightening the risk of endovascular infection, particularly arteritis [7]. The mortality rate associated with endothelial infection is estimated at 69% [2].

*S. enterica*-induced cardiovascular infections are uncommon, and their clinical characteristics, prognosis, and optimal therapeutic approach remain incompletely defined [2,4]. Unlike most gram-negative bacteria, *S. enterica *demonstrates a unique ability to adhere to damaged endothelial cells, particularly within the heart and arterial walls [2]. *Salmonella* endocarditis is a highly invasive and destructive infection that often leads to valve perforation, atrioventricular wall perforation, and leaflet rupture [2,4,7]. Although *Salmonella *enteritis is an unusual cause of endocarditis, representing only 0.01-2.9% of bacterial endocarditis cases, it is particularly concerning in prosthetic valve infections, with a mortality rate of 26.3-31.6% [2,4,10]. In both native and prosthetic valve endocarditis, the mitral and aortic valves are most commonly affected [2,4,7].

The most typical clinical findings in *Salmonella *endocarditis include fever (100%), heart murmurs (40%), heart failure (30%), central nervous system embolization (13%), pericarditis (10%), and heart block (3%) [2]. Recurrent bacteremia is a critical diagnostic indicator of endocarditis [2]. Imaging diagnosis generally relies on transthoracic or transesophageal echocardiography, with additional modalities like PET scans or cardiac MRI often required for a definitive diagnosis [2].

Indications for cardiac surgery in patients with invasive NTS disease include the presence of an implantable electronic device, vegetation greater than 10 mm, or peri-annular abscesses [10]. While prosthetic valve involvement, although less common, lacks a formal surgical indication, most patients ultimately require therapeutic intervention [4,10].

*Salmonella* UTIs are not always indicative of severe complications and may result from direct invasion through the urethra, especially in women [8]. Despite this, patients often have underlying risk factors, such as diabetes mellitus, immunodeficiency (including HIV infection, splenectomy, or malignancy), or anatomical abnormalities of the urinary tract [3,8]. Third-generation cephalosporins and fluoroquinolones are typically preferred due to their excellent tissue penetration, potent bactericidal activity, and efficient urinary excretion as active drugs [3].

To minimize the risk of treatment failure and recurrent infection, particularly in high-risk patients, prolonged antibiotic therapy is often recommended despite limited evidence regarding the optimal duration [3,8]. Although the infection is often manageable, long-term outcomes can be poor, with reported mortality rates reaching 22% [3].

Osteomyelitis, although rare, is a significant complication in patients with sickle cell anemia or thalassemia, contributing to substantial morbidity and mortality [9]. It is also observed in individuals with diabetes mellitus, systemic lupus erythematosus, lymphoma, cardiovascular and hepatic diseases, and those receiving corticosteroid therapy [9]. Osteomyelitis typically affects the diaphyseal regions of long bones, lumbar vertebrae, tibia, and radius [9].

Vertebral osteomyelitis presents a significant therapeutic challenge, with most studies supporting a two-month course of antibiotic therapy [9]. Selecting antibiotics with effective bone penetration is essential for optimal treatment outcomes [11]. For patients who do not respond to initial treatment or develop local complications, prolonged antibiotic therapy and surgical debridement should be considered [9].

As previously reported, the low prevalence of this disease complicates therapeutic regimens, requiring guidance based on local resistance patterns and the specific condition of each patient [1,4,6]. Due to increasing fluoroquinolone resistance, ceftriaxone is considered the first-line treatment [7]. However, resistance to ceftriaxone is also emerging [7]. Antibiotic therapy should last at least six weeks for surgical patients, with possible prolongation in non-surgical cases, those with persistent bacteremia (e.g., retained prosthetic material), or those with additional infectious foci like osteomyelitis [7].

We did not find any cases in the literature with such extensive involvement, which made the management of this patient even more complex. A multidisciplinary approach was essential, with a joint decision on the timing of antibiotic therapy and close monitoring of the evolution of the different sites of involvement by various specialties.

## Conclusions

Invasive *Salmonella* disease is a rare yet severe condition for which optimal management remains uncertain. Clinicians should consider invasive *Salmonella *infection in patients presenting with a history of diarrhea followed by persistent fever, bacteremia, and secondary focal infections such as osteomyelitis, endocarditis, and renal abscess. Remarkably, no previous reports in the literature describe invasive *Salmonella *disease with such extensive multisystem involvement as observed in this patient. The severity of the skeletal infection was particularly concerning, and due to its anatomical location, surgical intervention was deemed inappropriate. Additionally, the cardiac involvement posed a significant risk of necessitating valve replacement to achieve adequate disease control.

This case highlights the growing incidence of fluoroquinolone-resistant NTS, underscoring the importance of carefully selecting antibiotic therapy, particularly for high-risk patients and those with atypical disease progression. Despite the severity of the condition, the patient has shown favorable clinical progress and is currently under multidisciplinary outpatient follow-up.
